# Residue dynamics and dietary risks of diflufenican and flufenacet in wheat using field experiments and model simulations

**DOI:** 10.1002/ps.70702

**Published:** 2026-03-05

**Authors:** Nannan Pang, Xinze Liu, Peter Fantke, Qi Zhang, Liyuan Liu, Qiaozhen Chen, Qiyu Gong, Jiye Hu

**Affiliations:** ^1^ Lab of Pesticide Residues and Environmental Toxicology, School of Chemistry and Biological Engineering University of Science and Technology Beijing Beijing PR China; ^2^ substitute ApS Copenhagen Denmark; ^3^ Department for Evolutionary Ecology and Environmental Toxicology Goethe University Frankfurt am Main Germany; ^4^ Department of Environmental Sciences, College of Agriculture and Environmental Sciences University of South Africa Roodepoort South Africa; ^5^ Guobiao (Beijing) Testing & Certification Co., Ltd Beijing PR China

**Keywords:** dynamiCROP, human health, liquid chromatography‐mass spectrometry, pesticides, plant uptake

## Abstract

**BACKGROUND:**

Pesticide usage and related food safety and human health concerns have received great attention worldwide. The phenyl ether and oxyacetamid herbicides diflufenican and flufenacet are widely used in wheat production in China and beyond to control grasses and broad‐leaved weeds. We hence assessed their residue behaviors in wheat production and evaluated related dietary risks for humans.

**RESULTS:**

Comparing field trials and an established method of QuEChERS (quick, easy, cheap, effective, rugged and safe) liquid chromatography tandem mass spectrometry (LC–MS/MS) with simulations using a dynamic plant uptake model gave consistent residue results in wheat for both diflufenican and flufenacet. Residue levels were consistently below their respective maximum residue limits in China. Dissipation half‐lives for diflufenican were in the range 7.4–16 days in straw and 11–43 days in soil, whereas dissipation half‐lives for flufenacet were on average somewhat shorter, at 1.7–3.3 days in straw and 9.8–38 days in soil. Combining residues with human intake estimates allows for assessing related dietary exposure and risk. Chronic and acute risk quotients for both pesticides indicated acceptable dietary risk for consuming wheat products with respect to using diflufenican and flufenacet at recommended doses.

**CONCLUSION:**

Combining field trials with simulations from a dynamic plant uptake model allows for an effective and efficient evaluation of pesticide residues and estimates of related dietary risks for humans. Our approach can be extended to assess additional pesticides, crops and regions to foster safer pesticide use practices. © 2026 The Author(s). *Pest Management Science* published by John Wiley & Sons Ltd on behalf of Society of Chemical Industry.

## Graphical abstract

Combining field trials with model simulations allows for an effective and efficient evaluation of residues of herbicides diflufenican and flufenacet in wheat and related dietary risks for humans.

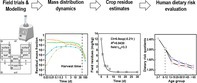

**ABBREVIATIONS**
ADI: acceptable daily intake
ARfD: acute reference dose
GC: gas chromatography
hF: harvest fraction
HR: highest residue level
iF: human intake fraction
JMPR: WHO/FAP Joint Meeting on Pesticide Residues
LC–MS/MS: liquid chromatography mass spectrometry
LOD: limit of detection
LOQ: limit of quantification
LP: large portion of food
QuEChERS: quick, easy, cheap, effective, rugged and safe
STMR: supervised trials median residue level
TF: translocation factor, Ue, unit weight
υ
: variability factor

## 1 INTRODUCTION

Pesticides are important to maintain high crop yields and ensure food security. However, potential intake of pesticide residues in harvested food crops can pose a risk to humans, whereas pesticides also can reach nontarget organisms, for example through run‐off and leaching.^1^, ^2^, ^3^, ^4^ Despite known harmful effects of pesticides,^5^ pesticide use is still an important aspect to control unwanted pests worldwide. Balancing crop yield and quality requirements, and impacts from pesticide usage is hence key for current agricultural production.

Wheat is a critically important global crop for human consumption. Herbicides have been widely used to enhance wheat yield. Among them, diflufenican and flufenacet have been used in combination in response to the increasing resistance toward pesticides in weed species.

Understanding dynamics of pesticide residues in different food crops is an important aspect for assessing related dietary risks for humans related to crop consumption.^6^, ^7^, ^8^ Pesticide residues can be determined *via* field trials, following different agricultural practices. However, conducting field trials for the large number of pesticides applied in various combinations across a wide variety of crops is labor‐intensive, time‐consuming and costly. Costs can be reduced by adopting cost‐efficient analytical techniques to assess residues obtained from field trials, such as the widely applied QuEChERS (quick, easy, cheap, effective, rugged and safe) liquid chromatography tandem mass spectrometry (LC–MS/MS).^9^, ^10^ Furthermore, field trials can be combined with mass balance modeling to provide additional insights into how the underlying processes influence residue dynamics, such as leaching, volatilization, degradation and translocation of pesticides within crops.^11^, ^12^ Combining targeted field trials with environmental modeling is hence a viable option to quantify and consistently interpret the environment fate of pesticides and residue behaviors in the complex ecosystem.^13^, ^14^

A wide range of models which focused on cereals, vegetables, root crops and fruit trees have been developed and reviewed.^15^ Among these models, dynamiCROP provides transparency and flexibility for studying the complex pesticide‐crop‐environment system across a range of crops, and is specifically parameterized for assessing pesticide residues in wheat, based on dynamically solving the underlying mass balance for a set of interconnected plant and environmental compartments.^15^, ^16^, ^17^, ^18^ Using a series of first‐order linear differential equations, it considers the distribution, bioaccumulation, translocation, transformation and degradation in environments such as soil and air, and inside the crop components.^16^, ^17^ Relying on matrix algebra for solving the mass balance system, the model yields pesticide residues in the various environmental and plant components at varying harvest time, and translates residues in crop harvest into human intake of processed crop‐based food.^17^

Applying plant uptake models in different studies shows that mass balance models are an effective way of predicting pesticide behavior in different crop systems, and highlight that intake of pesticide residue in harvested crop compartments is an important human exposure pathway.^13^, ^19^, ^20^, ^21^, ^22^ The dynamiCROP model has been most frequently used to assess residues of various pesticides applied to wheat,^11^, ^15^, ^23^ tomato^16^, ^23^, ^24^ and lettuce,^16^, ^23^, ^25^ with additional uses on crops such as passion fruit, cucumber and cabbage.^26^, ^27^ There has been a continuous development of dynamiCROP, such as including additional crops (e.g. cucumber and cabbage),^25^ new compartments such as periderm,^28^ and the application to multiple pesticide applications over time.^29^

Although diflufenican and flufenacet are widely used in agriculture, their residues in wheat were mostly studied as part of large‐scale screenings without any focused analysis of their residue dynamics, the contribution of underlying processes to these dynamics, and related dietary risks for humans after a combined application specifically to wheat crops (see Supporting Information Section S1, Table S1, for details on available residue studies for these two pesticides). To address this gap, the goal of the present study is to combine field trials with model simulations as an effective method to assess residue dynamics and related dietary risk of two pesticides applied to wheat crops. In response to this gap, the present study aims at conducting a detailed analysis of the distribution of diflufenican and flufenacet after application to wheat, assess related residues in harvested crop components and evaluate the associated dietary risk for different human age groups. We thereby focus on the following specific objectives: (i) to implement field trials and a QuEChERS LC–MS/MS method to derive residues of diflufenican and flufenacet in wheat; (ii) to apply a dynamic plant uptake model to complement experimentally derived residues with model estimates; (iii) and to evaluate related dietary risks for humans in support of decision making for pesticide management and food security.

## 2 MATERIALS AND METHODS

### 2.1 Chemicals

Diflufenican (CAS NO. 83164–33‐4) and flufenacet (CAS NO. 142459–58‐3) substances were obtained from Beijing QinChenYiXin Co. Ltd (China). Other common reagents included: acetonitrile and formic acid (Thermo Fisher Scientific, Waltham, MA, USA), NaCl, MgSO_4_ and acetic acid (Beijing Chemical Reagents Company, China); and primary secondary amine (PSA) (40–60 μm) (Agela Technologies, Tianjing, China)). A 500 μg mL^−1^ solution of diflufenican and flufenacet in acetonitrile was used for the subsequent dilution using solvent and blank sample matrix, sequentially and separately.

### 2.2 Pesticide application field trials

In order to diversify the sample distribution and reflect different climate and soil conditions,^30^ field trials of pesticide application were carried out in open‐air conditions in China. Three sites were chosen, including Beijing (116.46° E, 39.92^o^N), Shandong (120.42° E, 36.58^o^N) and Anhui (116.93° E, 34.19^o^N). Soil pHs were 6.73, 7.32 and 6.79, respectively, and soil organic matters were 2.7%, 3.9% and 1.7%, respectively. The average temperature ranged from 21 °C to 24 °C in 2017. No agrochemical ingredients were used earlier that are similar to diflufenican and flufenacet.

A 30% suspension concentrate of diflufenican and flufenacet (1:2) was used, corresponding to 270–450 g active ingredient (a.i.) ha^−1^. In our dissipation experiment, we applied 675 g a.i. ha^−1^, and in the terminal residue experiment, we applied 675 and 450 g a.i. ha^−1^. Three replications were used for each treatment (50 m^2^). The untreated plot also was 50 m^2^ with a spray of clean water instead of pesticides. Different treatments were performed with a 1‐m spacing. Straw and soil samples at different intervals (2 h, 1, 3, 5, 7, 14, 21 and 30 days) were extracted for the dissipation trial. Straw, grain and soil samples on Day (D)63 were harvested for the terminal residue trial. Samples were stored at −20 °C and analyzed within 3 months.

### 2.3 QuEChERS LC–MS/MS procedures

First, 5 g soil, 5 g grain and 2 g straw were homogenized and pretreated by QuEChERS, separately. QuEChERS had two parts. In the extraction part, acetonitrile (10 mL), water (5 mL) and acetic acid (200 μL) were added with 1‐min vortexing. NaCl (1 g) and MgSO_4_ (4 g) were subsequently added with another 1‐min vortexing. After centrifugation, PSA (50 mg) and MgSO_4_ (100 mg) were added into the supernatant (1.5 mL) with 1‐min vortexing. After centrifugation, the resultant supernatant was filtered and analyzed.

A 1260 infinity & triple quadrupole LC–MS apparatus (Agilent Technologies, Santa Clara, CA, USA) was used in this work. The column was Agilent Poroshell 120 EC‐C18 (50 mm × 3.0 mm, 2.7 μm). The isobaric gradient was 0.2% formic acid (20%) and acetonitrile (80%). Other parameters were flow rate 0.4 mL min^−1^, temperature 40 °C and injection volume 5 μL. MS parameters included positive multiple reaction monitoring (details are provided in Table S2), capillary voltage 4000 V, nebulizer pressure 45 psi, drying gas (N_2_) flow 11 L min^−1^ and dry gas temperature 350 °C.

QuEChERS LC–MS was optimized and established for the simultaneous determination of diflufenican and flufenacet, validated by evaluating recoveries in all three matrices (soil, straw and grain), matrix and cleanup effects, and limits of quantification (LOQ). Validation methods and results are presented in Section S3. According to our method validation, the LC–MS analysis was within 1.7 min in matrices of soil, wheat grain and straw (Fig. S1), and showed acceptable recoveries (85%–103%; Fig. S2), matrix effect ranges (72.7 to 109%; Table S3) and linearity with correlation coefficients (*R* ^2^) > 0.99 and LOQs = 0.01 mg kg^−1^ in all matrices (Table S3). Thus, this QuEChERS LC–MS method using simple and common sorbents was suitable for determination of diflufenican and flufenacet in three matrices, with acceptable quantitation, satisfactory recoveries and LOQs.

### 2.4 Plant uptake model simulations

The dynamiCROP plant uptake model was used to simulate the uptake and mass distribution of the considered pesticides in the wheat ecosystem.^15^, ^16^, ^17^, ^18^ In our study, the pesticides of diflufenican and flufenacet, the wheat crop as well as the applied pesticide amount were specified in the model. The model was parameterized for pesticide application to wheat by defining appropriate wheat crop parameters (e.g. transpiration, crop component properties such as stem height and water content, leaf area index and pesticide interception area upon application) and environmental characteristics (e.g. soil depth and organic carbon content, and height of air column above the crop). These general parameters were adopted from previous studies.^15^ Based on solving the mass balance for each pesticide in the wheat–environment system, the distribution dynamics of diflufenican and flufenacet mass across considered environmental and wheat crop compartments could be simulated, including how each environmental fate process contributes to pesticide‐specific mass distributions in these compartments.

### 2.5 Deriving residue dynamics in wheat

Residue measurements from our field trials gave results at different time intervals after pesticide application. From these results, we could determine the dissipation behavior, assuming first‐order kinetics^31^:

(1) $$C_{t}=C_{0}\;e^{-\mathrm{kt}}$$

where C is the pesticide concentration at different times after boom sprayer application, t is the time and k is the first‐order rate constant. From k, we derive the dissipation half‐life according to $t_{1/2}=\ln\left(2\right)/k$, as an important parameter in plant uptake models that often drives overall residues across crop components. Half‐lives along with other relevant physicochemical substance parameters (see Table S1) for both considered pesticides were used as input for the dynamiCROP model simulations. Based on these inputs, the model yields mass distributions for each pesticide over time across the various crop components and environmental compartments. Details of the underlying mass balance solution and considered environmental and crop processes (e.g. root uptake, within‐crop translocation, volatilization) are described elsewhere.^13^, ^14^, ^15^, ^16^

Harvest fractions (hF) were then calculated as the ratio between the residual mass at harvest and the pesticide mass applied to the crop.^13^

Once entering plants, pesticides were transferable within the plant. A chemical can transfer from root to shoot, expressed by the translocation factor (TF).^32^, ^33^ This is the ratio between the concentrations in shoot and root; in this study, the shoot concentration corresponded to the sum of those in stem and leaf:

(2) $$\mathrm{TF}=\frac{C_{\text{stem}}+C_{\text{leaf}}}{C_{\text{root}}}$$

### 2.6 Deriving dietary risks

Pesticide residues in the harvested wheat components were used as a starting point for estimating related dietary risks for humans for both assessed pesticides. We considered both chronic and acute dietary risks in our study. Legislation such as World Health Organization (WHO) / Food and Agriculture Organization of the limited States (FAO) Joint Meeting on Pesticide Residue (JMPR) give important parameters after careful investigation, such as acceptable daily intake (ADI), acute reference dosage (ARfD) (see Table S4). Detailed calculations are provided in Section S2. In brief, for the long‐term evaluation, the chronic risk quotient RQ_C_ was calculated according to Eqns (3) and (4):

(3) $$\text{NEDI}=\sum_{i}\left(\text{STMR}\times F\;\right)/\text{body weight}$$

(4) $${\mathrm{RQ}}_{C}=\text{NEDI}/\mathrm{ADI}\times 100\%$$

where NEDI is the average national estimated individual daily intake, corresponding to the sum of the appropriate residue in all food taken in by a human of a specific human group, STMR_i_ is the standard median residue (mg kg^−1^) and F_i_ refers to food consumption by a human of a specific human group. Body weight is the average value for a human group.

Likewise, the acute risk quotient RQa was for the acute risk evaluation and calculated according to Eqns (5) and (6):

(5) $$\text{NESTI}=\left[\frac{\mathrm{HR}\times \mathrm{LP}\left(\mathrm{no}\;\mathrm{Ue}\right);\mathrm{Ue}\times \mathrm{HR}\times \upsilon +\left(\mathrm{LP}-\mathrm{Ue}\right)\times \mathrm{HR}\left(\mathrm{Ue}<\mathrm{LP}\right);\mathrm{HP}\times \mathrm{LP}\times \upsilon \left(\mathrm{Ue}>\mathrm{LP}\right)}{\text{body weight}}\right]$$

(6) RQa=NESTI/ARfD×100%

Compared with NEDI, NESTI is the short‐term (ST) value, which estimates the maximum pesticide intake acutely. HR is the highest residue in field trials. Different foods have their unit weight (Ue) (if existed), the variability factor (ν) for intake correction (with large Ue) and the large portion (LP) (97.5% of the daily consumption) of food intake in one meal.

The resulting RQc and RQa are chronic and acute risk quotients, respectively, via pesticide residue intake from crop consumption by humans. Risk quotients <1 are referred to be of ‘acceptable risk’ for humans.

## 3 RESULTS AND DISCUSSION

### 3.1 Experimentally derived residue levels of diflufenican and flufenacet

After pesticide application, absorption, volatilization, hydrolysis and degradation occurred simultaneously. Field dissipation information assisted in better understanding of the pesticide behaviors leading to residues that humans were exposed to. There were plenty of samples to diversify the whole sample composition which facilitated the representation of the field situation as real as possible. For example, kinetic samples were collected in not only three representative districts with different climate and soil properties in China, ranging from the north to the south but also different across time intervals. Residues in soil and straw samples were analyzed and the first‐order kinetic fitted well. Table S5 shows the kinetic equations of diflufenican and flufenacet with *R* ^2^ in the range of 0.84–0.99. Their dissipation half‐lives also are shown in the Table S5.

In straw samples, diflufenican and flufenacet showed not only high residue, but also fast degradation. Because there has been no dissipation reported in literature, each residue in straw at different time interval is listed in Table S6 expressed as the dissipation ratio compared with the initial deposit. For diflufenican, the initial concentration was 4.2–10 mg kg^−1^ in straw, almost four‐fold higher than in soil (Table S5). Diflufenican dissipated faster in straw according to the half‐life of 16 days (maximum) compared with 43 days (maximum) in soil. For flufenacet, the situation was similar; the highest initial residue and the longest half‐life in straw were 17 mg kg^−1^and 3.3 days, respectively, compared with 4.4 mg kg^−1^ and 38 days in soil. In soil samples, both pesticides showed persistence with >10 days of half‐lives in soil. In some sites, their half‐lives in soil could be as high as 43 days. Besides, the degradation of flufenacet was faster in straw than diflufenican, almost five folds (3.3 days *versus* 16 days) (Table S5). Diflufenican persisted longer in straw and soil. For example, according to Table S6, the dissipation ratio of diflufenican and flufenacet reached 49.4% and 70.0% on 30 days in Shandong (site B), respectively. The original ratio of 1:2 (less diflufenican) was thus appropriate if maximizing the pesticide effect while maintaining the shortest pre‐harvest interval (PHI).

In this study, 30% suspension concentrate real formulation of diflufenican and flufenacet (1:2) was applied following good agricultural practice in field trials. Terminal samples (soil, grain and straw) were collected not only in three different districts in China, but also with two different application dosages. The diversity of terminal samples guaranteed the correct residue evaluation in field conditions. In particular, residue levels in edible parts such as grain were paid more attention to considering the potential food safety risk. In this study, residues of both two herbicides were undetectable in edible wheat grain (< LOQ) at the harvest time. Considering MRLs of diflufenican (0.05 mg kg^−1^) and flufenacet (0.5 mg kg^−1^) in China, our method was suitable for the terminal residue monitoring in real samples. The residue levels of these two could also give useful information and suggestions for other guidelines, such as the Codex Alimentarius Commission (CAC).

### 3.2 Modeled evolution of pesticides

**Figure 1:**
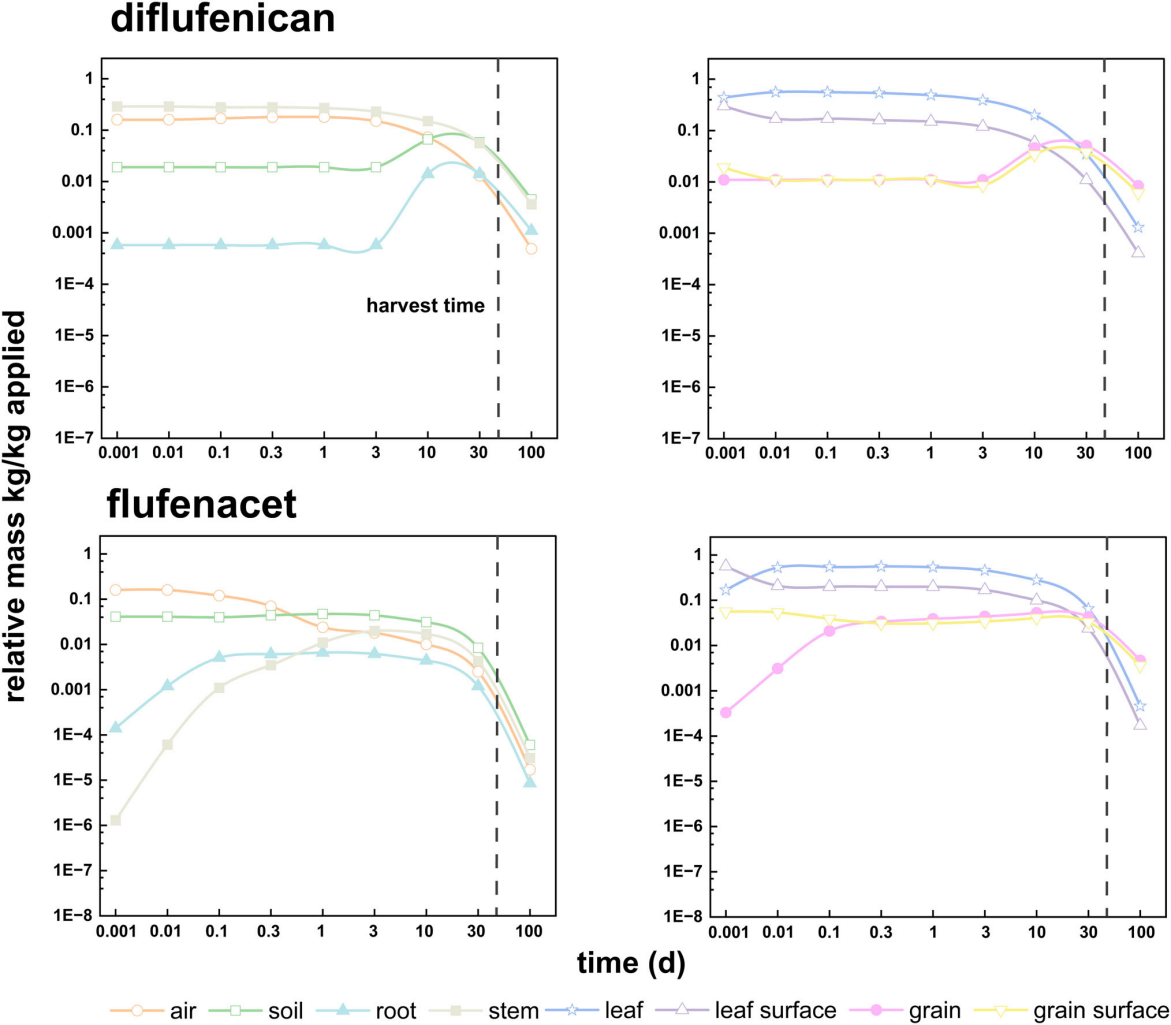
Evolution of masses of diflufenican and flufenacet in the wheat ecosystem with main driving compartments.

In dynamiCROP, there were eight compartments considered: air, soil, leaf surface, grain surface, leaf, grain, stem and root. The former four compartments received pesticides initially (i.e. source compartments for pesticide application). After application and initial distribution, the latter four compartments received pesticides as residues by chemical diffusion, advective transport and degradation processes. All were modeled relying on chemicals, crops and environmental data. Figure 1 shows the mass evolution of diflufenican and flufenacet. Different underlying pesticide distribution mechanisms dominated different periods since the pesticide application. For example, diffusion and transport processes largely explained the initial dynamics of pesticides right after application. Accumulation and degradation, by contrast, explained the dynamics of pesticide distribution in the longer term and close to crop harvest.

Shortly after application, both pesticides quickly entered leaf, stem, root and grain, and accumulated. Then, pesticide masses in leaf, grain interior, stem and root increased. There existed a maximum for each compartment (Fig. 1). The maximum relative masses ranged from 6.6 × 10^−3^ kg kg_applied_^−1^ in root after 1 day for flufenacet to 0.56 kg kg_applied_^−1^ in leaf at 0.1 day (diflufenican) and 0.25 day (flufenacet), respectively. After the maximum peak, pesticide masses began to decrease more or less exponentially owing to the increasingly relevant degradation processes as well as growth dilution. In the long term, the residence time of two pesticides (16.27 days for diflufenican and 15.66 days for flufenacet in grain) illustrated that the long‐term dynamics were driven by the grain compartment (see Table S7).

### 3.3 Modeled residue distributions

**Figure 2:**
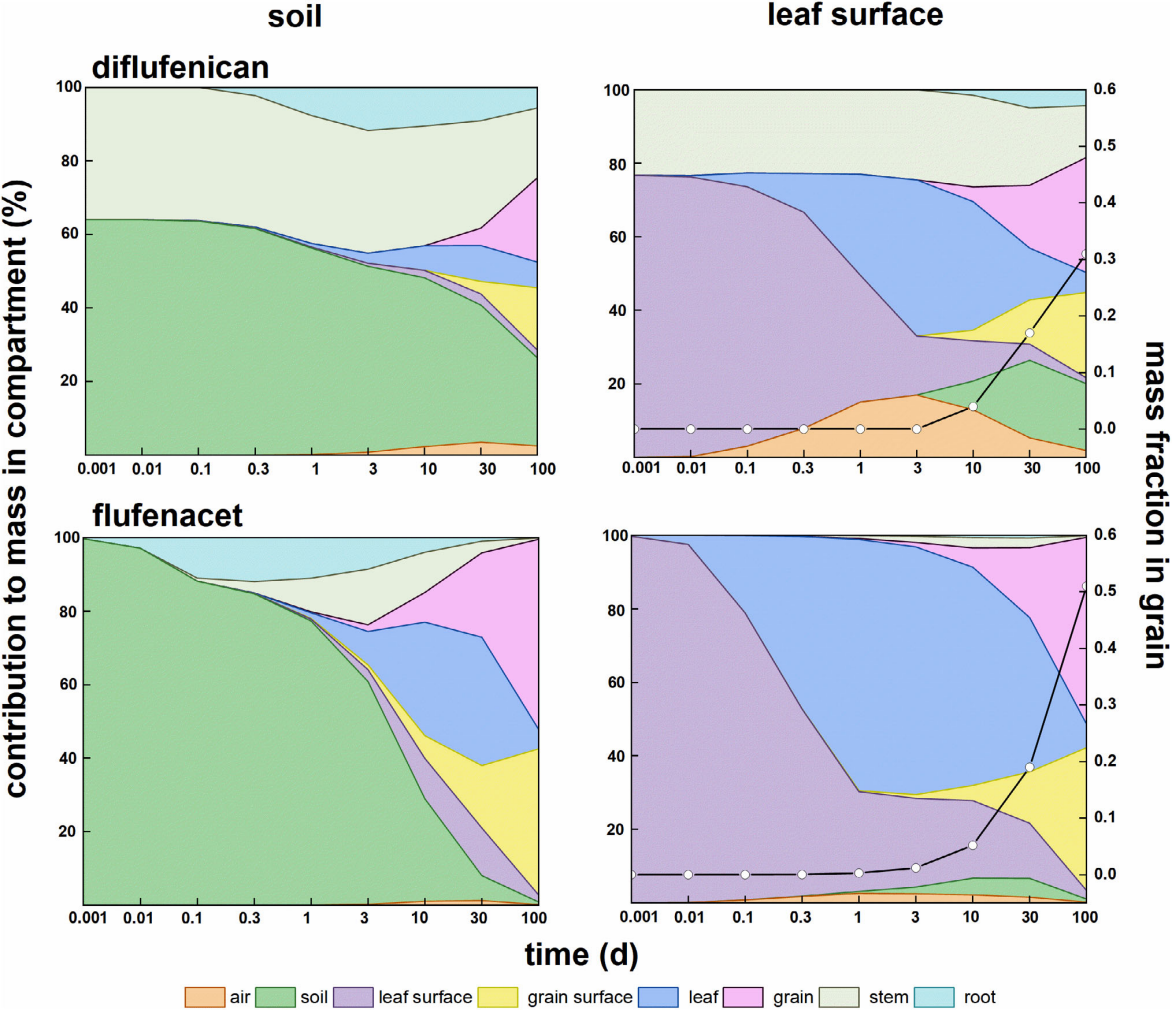
Evolution of whereabouts of applied diflufenican and flufenacet ending up as residue in wheat after soil and foliar spray application. Black lines in right‐side plots show the evolution of overall fraction of applied pesticide mass in the grain.

There were two main pesticide application methods in our study: foliar and soil spray. Different application methods corresponded to different pesticide distributions. To illustrate the pesticide residues with different applications, Fig. 2 shows the distribution pattern of diflufenican and flufenacet with soil and foliar spray methods, respectively. Figure 2 shows the whereabouts of the pesticide, when the pesticide was applied exclusively to a single compartment, namely soil or leaf. The pesticides experienced not only degradation, but also diffusion and advective transfers in wheat. However, at present, most studies could explain the pesticide distribution in tissues only cultivated in the growth medium and artificial greenhouse conditions throughout the determination of pesticides in different tissues.^32^, ^33^, ^34^, ^35^, ^36^ By contrast, Fig. 2 gives the distribution pattern and ratio in different compartments with soil and foliar spray methods without laborious experiments.

Different pesticides and application methods corresponded to specific distribution characteristics. For the soil application, for diflufenican and flufenacet, most residues still stayed in soil within 10 days after the application. From then on, residues began to distribute into eight compartments. For the foliar spray application of diflufenican, residues in the grain surface and the grain were highest. With the growth of grain, the grain residue became dominant. It indicated the easy translocation to grain for diflufenican. For flufenacet, residues in the leaf surface were highest. With time going by, the leaf residue became dominant.

With foliar spray, the fractions in grain relative to the total residue mass for diflufenican and flufenacet are shown in Fig. 2. Both diflufenican and flufenacet showed an increasing residue trend. In the initial period of 10 days, the fractions in grain for both diflufenican and flufenacet were small but then increased with time. The fractions in grain of diflufenican and flufenacet were close to each other, 0.17 *versus* 0.19 at D30. However, the fraction in grain of flufenacet reached 0.51 at D100 with a sharper increasing trend compared with the increase to 0.31 of diflufenican at D100. The increasing amplitudes between D10 and D30 and between D30 and D100 were both in an increasing order of diflufenican < flufenacet. However, the increasing trend between D100 and D300 was an increasing order of flufenacet < diflufenican.

Flufenacet showed a greater mobility in this application, which was supported by the above‐mentioned dissipation half‐life comparison between diflufenican and flufenacet wheat straw (16 days *versus* 3.3 days). Usually, water soluble pesticides (log *K*ow < 2) led to a quasi‐equilibrium of faster uptake compared with more lipophilic pesticides (log *K*ow > 3).^33^ Furthermore, the translocation ability of pesticides increased with the decrease of hydrophobicity and *K*ow. Thus, considering their log *K*ow (4.2 *versus* 3.2), flufenacet was more mobile than diflufenican. Additionally, the fractions in grain could be compared between different pesticides. For example, fluroxypyr showed a fraction in grain of 0.6 at D30.^11^ Thus, the mobility of diflufenican and flufenacet was higher than that of fluroxypyr whose log *K*ow is −1.24. Thus, different fates of different pesticides could be compared between each other.

### 3.4 Contribution of compartments to residue dynamics

**Figure 3:**
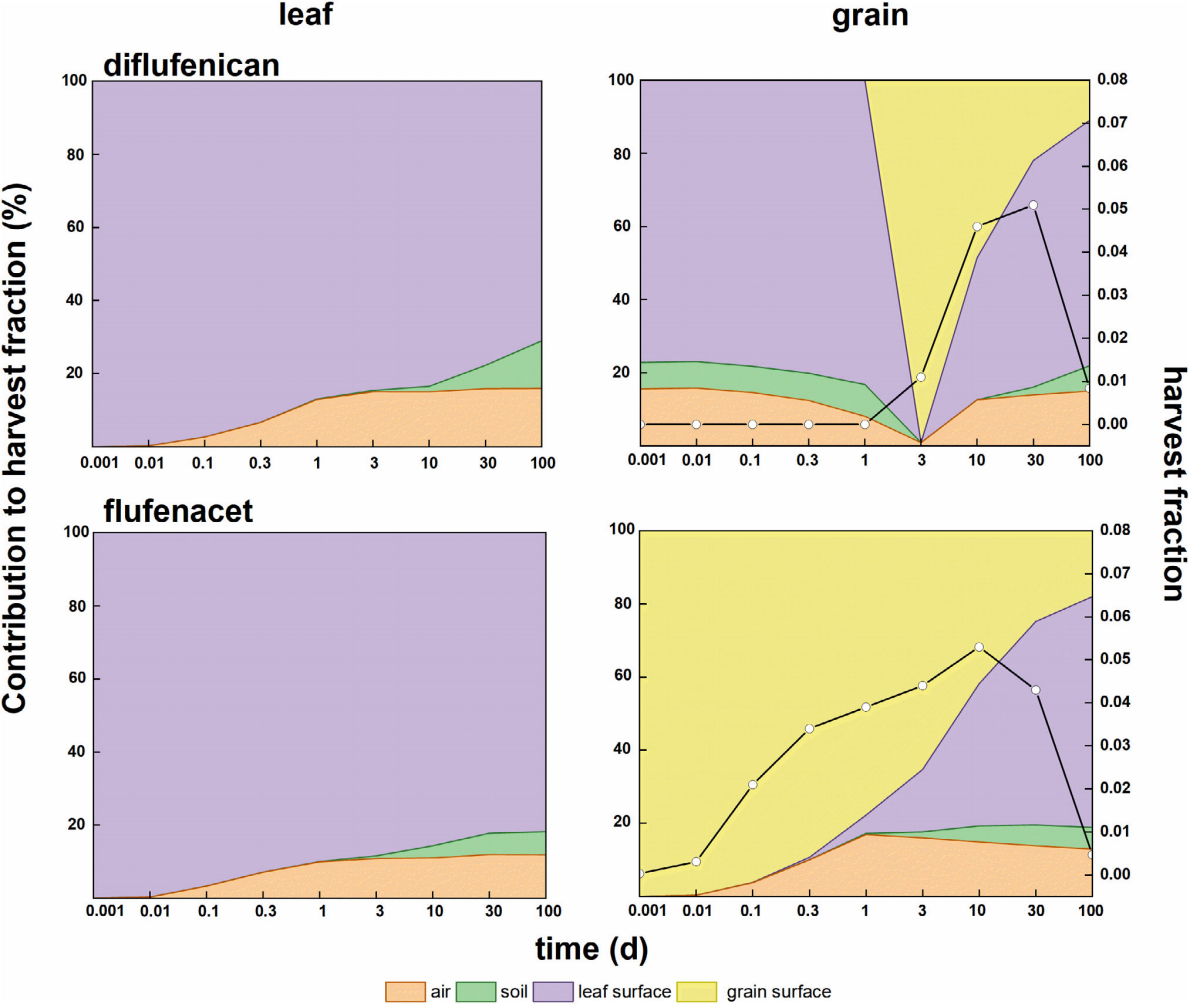
Evolution of contribution of initial pesticide in air, soil, leaf surface and grain surface to masses in leaf and grain. Black lines in right‐side plots show the evolution of the harvest fraction, hF, in the grain.

Air, soil, leaf surface and grain surface belonged to source compartments that received the applied pesticide initially. Residues then diffused, transferred, degraded and finally entered the crop interior. Residues in edible and nonedible parts brought in different perspectives in food safety and pesticide management. The former was compared with MRL. The latter was used to calculate half‐lives of pesticides in wheat. After application, pesticides were distributed in source compartments according, for example, to application method, time, crop properties, and weather. Then, the contribution of each source compartment to crop residues at different time could be obtained by dynamiCROP as shown in Fig. 3. This shows the four source compartment contributions to residues in leaf and grain for the two pesticides. The contribution was a dynamic process after the pesticide application.

In the leaf compartment, the main contribution was from the leaf surface. For diflufenican and flufenacet, the contribution of source compartments shows similar evolutions. Although air and soil provided some pesticide residue, the leaf surface compartment contributed overwhelmingly.

In the grain compartment, the situation was more complex. For diflufenican, initially, the contribution of leaf surface was high, indicating that the residue in grain was mainly transferred from the leaf surface compartment. Afterwards, in the middle term, with the growth of the grain, the grain surface's contribution has become dominant since D3 after application. Then the leaf surface took over again quickly. At D30, the contribution of the leaf surface exceeded those of other source compartments. For flufenacet, initially, the grain surface dominated until D3 after application. During the D3–D10 period, the leaf contribution dominated. With time, the soil contribution played a greater role as main sink in the longer term.

Figure 3 also presents the harvest fractions (hFs) in grain. The peak values of hFs for diflufenican and flufenacet (see Fig. 3 and Table S7) occurred between D10 to D30. From the practitioner's view, it is thus better to avoid foliar spray, namely the leaf surface contribution, to guarantee relatively low residues of diflufenican and flufenacet. In addition, the harvest time should not fall within the time frame with the highest hFs to meet the requirements of pesticide management.

### 3.5 Comparison between modeled and measured residues

**Figure 4:**
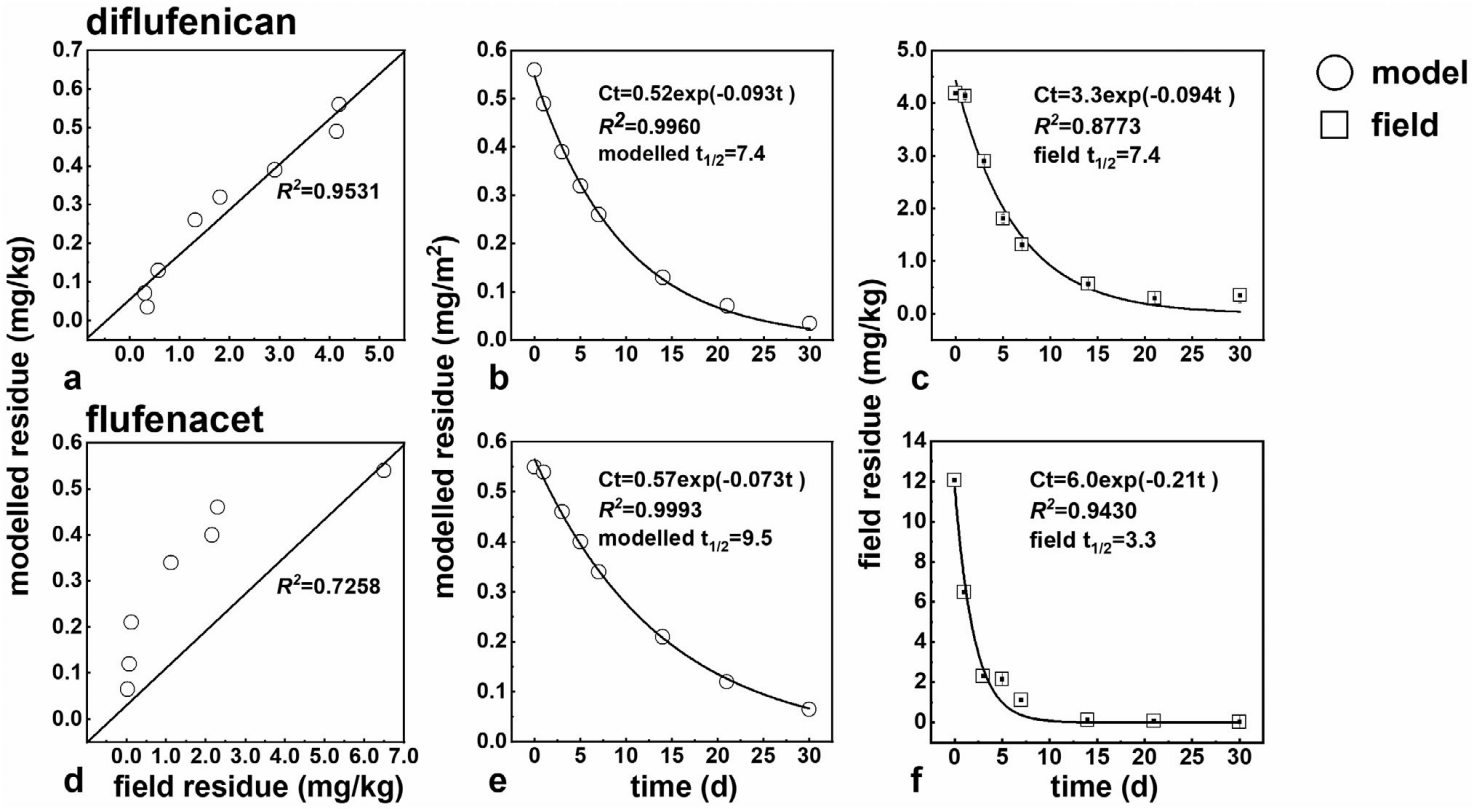
Modeled and field (in China) residues and dissipation kinetics in wheat straw of diflufenican in (a, b, c) and flufenacet (d, e, f) determined by QuEChERS LC–MS/MS.

In this work, 30% suspension concentrate of diflufenican and flufenacet (1:2) was applied *via* foliar spray in field trials. The applied dosage was low, in the magnitude of mg m^−2^. Diflufenican and flufenacet were determined in grain and straw by QuEChERS LC–MS/MS. Based on the QuEChERS LC–MS/MS method, residues were obtained and compared with modeled ones at different intervals (1–21 days) [Fig. 4(a), (d)]. Meanwhile, the applied model gave residues on different time intervals [Fig. 4(b), (e)]. Results from both were compared with each other. From Fig. 4(a), (d), it can be seen that the modeled and the measured residues fitted relatively well with the corresponding *R* ^2^ of 0.95 and 0.73 for diflufenican and flufenacet, respectively. In the model, the first‐order kinetics perfectly fitted for straw with *R* ^*2*^ higher than 0.99 for diflufenican and flufenacet [Fig. 4(b), (e)]. In the field trials, the dissipation kinetics also were good, with *R* ^*2*^ of 0.88 and 0.94, respectively [Fig. 4(c), (f)]. Thus, for diflufenican, the half‐life of the model and the field trial agreed well (both 7.4 days). For flufenacet, the modeled half‐life was 9.5 days, compared with the field one of 3.3 days. The deviation might come from the residues, which were determined around the LOD by QuEChERS LC–MS/MS owing to the low applied dosage in the field.

### 3.6 Translocation factors, harvest fractions and human intake fractions

Translocation factors illustrate the pesticide transferring between tissues of plants.^35^ Usually, it is the ratio between pesticide residue in straw to root, illustrating the transferring of pesticides from underground to aboveground tissues. It is difficult to realize owing to the need for laborious experiments, calling for accurate pesticide quantitation in different tissues using the soil application of pesticides. Thus, only plants cultivated in growth medium could provide TF values to evaluate the upward translocation ability of the pesticide.^33^, ^34^, ^35^ Our modeling approach could give the simulated TF values of pesticides in plants in a dynamic way. For example, TFs of diflufenican and flufenacet with the soil application are shown in Fig. S3. TFs changed with the growth of wheat, and those of flufenacet were larger than those of diflufenican. The TF of diflufenican decreased initially and sharply, then increased to 4.5, and kept constant around 100 days. The maximum TF of flufenacet was around 57, which occurred close to the time of harvest. Liu *et al*. (2023)^36^ demonstrated that TF > 1 verified the preferential upward translocation from underground tissues to aboveground tissues, further indicating the pesticide transfer to food. Other contributing factors to the transport of pesticides from roots to shoots included solubility, lipid content and related *K*ow. In particular, log *K*ow has been reported to be negatively correlated with TFs;^37^, ^38^ the smaller log the *K*ow, the larger the TF, indicating the concentration tendency of more hydrophobic pesticides in roots.

Human exposure is a consequence of pesticide residues in consumed food crop components. However, field trials are less straightforward to determine these values owing to the need for laborious experiments and high costs, or owing to undetectable terminal residues because of the low applied dosage. Our modeling approach, hence, provides an additional way to evaluate the pesticide intake of humans, namely the maximum residue (Table S7). For diflufenican and flufenacet, the maximum residue occurred on D20 and D13, respectively. The residence times in grain were relatively long, at 16.27 days and 15.66 days for diflufenican and flufenacet, respectively. Additionally, the model directly yielded two fractions: hF and iF. hF illustrates the pesticide residue fraction at crop harvest time. After harvest, food crops experience food processing, which further reduce the intake pesticide residue. This leads to iF which is the intake pesticide residue fraction ultimately consumed in food by humans. The processing factor from wheat grain to bread was determined at 0.33.^15^ According to Table S7, modeled hFs for diflufenican and flufenacet ranged from 6.6 × 10^−3^ in root to 0.56 in leaf. Maximum hFs in edible grain were ≈5.5 × 10^−2^ kg_in harvest_ kg_applied_^−1^. Besides, modeled maximum iFs for diflufenican and flufenacet were around 1.8 × 10^−2^ (Table S7). Although these two pesticides had different applied dosage and different dissipation half‐lives, they had similar iFs in the range of 1.8 × 10^−3^ to 1.9 × 10^−3^ kg_intake_ kg_applied_^−1^.

### 3.7 Dietary risk assessment

Dietary risk assessment is explained in Part 2.6 and the Supporting information, Section S2. RQc and RQa were used for chronic and acute dietary risk assessment, respectively. Accordingly, standard median residue (STMR) and ADI (0.2 mg kg^−1^ for diflufenican, 0.005 mg kg^−1^ for flufenacet; Table S4) were used in the calculation of RQc. Likewise, the HR and ARfD were used in the calculation of RQa. No ARfD has been set for diflufenican considering its low toxicity and 0.017 mg kg^−1^ bw of ARfD is set for flufenacet owing to its relatively high toxicity (Table S4). Up to now, there is insufficient information available regarding dietary risk assessment for diflufenican and flufenacet.

Dietary composition and population characteristics are different for countries and districts. In China, the corresponding registered crops included wheat, rice and corn which were classified as cereals for the two considered pesticides (Table 1). Considering the importance of cereals in the Chinese diet, the corresponding data of body weight, the food intake and the large portion (LP) were used in the calculation (63 kg, 138.5 g and 732.96 g person^−1^). Field trials directly gave residues such as STMR and HR, leading to the calculation of RQc and RQa. In the long‐term risk assessment, for diflufenican, STMR in trials of 0.01 mg kg^−1^ was used. The resulting RQc for diflufenican was 0.01% as shown in Table 1. For flufenacet, the situation was similar, with a RQa of 0.44% (Table 1). Acute risk assessment of diflufenican could be neglected considering its unnecessary ARfD according to JMPR. For flufenacet, all residues were below detection limits (<0.01 mg kg^−1^), thus HR of flufenacet was maximized to this value. The corresponding RQa of flufenacet was 0.71%. Additionally, considering the body weight of 16.1 kg for children younger than 6 years of age as well as their food consumption, namely, LP (25.8 g), the RQa of flufenacet for children was 0.09%.

**Table 1:** Long‐term and short‐term dietary risk assessment of diflufenican and flufenacet in China for a person of average body weight (63 kg)

		Long term evaluation^†^				Short term evaluation^‡^					
Pesticide	Registered crops	ADI (mg kg^−1^ bw)	Modeled		Field		ARfD (mg kg^−1^ bw)	Modeled		Field	
			STMR (mg kg^−1^)	RQc %	STMR (mg kg^−1^)	RQc %		HR (mg kg^−1^)	RQa %	HR (mg kg^−1^)	RQa %
Diflufenican	cereal (wheat, rice)	0.2	0.011	0.012	0.01	0.011	_^§^	0.011	_^§^	0.01	_^§^
Flufenacet	cereal (wheat, corn)	0.005	0.022	0.97	0.01	0.44	0.017	0.022	1.58	0.01	0.71

^†^ ADI, acceptable daily intake; STMR = supervised trials median residue levels; Calculation refers to Eqns (3) and (4).

^‡^ ARfD, acute reference dosage; HR = the highest residue; Calculation refers to Eqns (5) and (6).

^§^ Toxicity is low.

**Figure 5:**
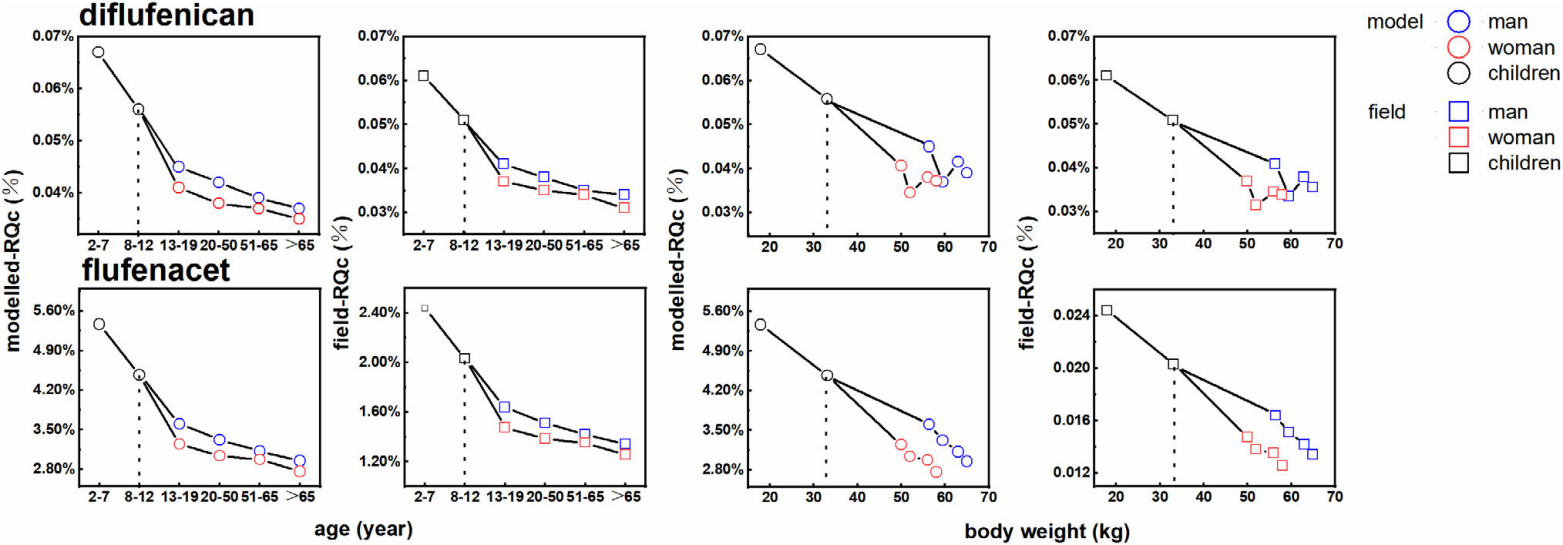
Chronic dietary assessment of diflufenican and flufenacet for different groups of people in China.

Different human groups have different sex, ages, body weights, dietary structures and preference. Hence, our dietary risk assessment focused on diversified human groups, based on not only body weight, but also dietary structure. For example, children, the elderly and woman, especially when pregnant, might be more vulnerable to the potential pesticide threat from diet. Table S8 and Fig. 5 provide detailed evaluations for different age groups. Generally, the dietary risk of different human groups decreased with increasing age for diflufenican and flufenacet from the 2–7‐year‐old group to the >65‐year‐old group. Both pesticides demonstrated similar long‐term risk patterns. Generally, the chronic dietary risk of flufenacet was higher. Children aged 2–7 years were faced with the highest chronic dietary risk (0.06% for diflufenican and 2.44% for flufenacet, respectively, based on field data), calling for urgent attention in pesticide management and food safety. Men and women usually faced similar risk (0.03–0.04% for diflufenican for both sexes and 1.34–1.64% *versus* 1.26–1.48% for flufenacet for men and women, respectively, based on field data). Overall, children were at somewhat higher risk related to the consumption of pesticide residues.

Model results also could yield the maximum residue (Table 1). Maximum residues were derived from diflufenican (0.01 mg kg^−1^) and flufenacet (0.02 mg kg^−1^), respectively. To maximize the risk, STMR used the value of HR in the evaluation. The modeled RQc values for diflufenican and flufenacet were 0.01% and 0.97%, respectively. The RQa for flufenacet was 1.58% based on model results. For the general population, the modeled and field results were comparable with each other, yielding both an acceptable risk (<1). Additionally, the modeled RQc trends were comparable with the field ones, including their changes along with age, body weight and sex (Fig. 5 and Table S8).

Pesticide properties are important aspects influencing potential dietary risk. Field trials and modeling of diflufenican and flufenacet provided useful information in understanding residues and related risk, and illustrated that neither pesticide applied to wheat showed any unacceptable risk for human health in China when used according to good agricultural practices, not considering simultaneous intake of other pesticides *via* wheat or other food crops.

## 4 CONCLUSIONS

A combined application of diflufenican and flufenacet which have been widely used in agriculture were analyzed by field experiments as well as a dynamic plant uptake model. Their comparison and combination enabled fast and reliable data processing about pesticide residue evaluation. Results from modeled residues and from confirmatory LC–MS. QuEChERS‐LC–MS/MS enabled the reliable determination of diflufenican and flufenacet in wheat–environment systems, and provided dissipation and terminal residue information in different field districts. The field trials and modeling could hence both be used to support dietary risk assessment. Our results demonstrated that diflufenican and flufenacet were safe to use with the recommended dosage, considering their residue and acceptable dietary risk. Additionally, the underlying modeling approach helped to predict pesticide fates as well as their translocation behaviors cost‐ and time‐efficiently. Thus, this approach is useful for researchers, regulators and practitioners when studying pesticide half‐lives and possible dietary risks for humans, and could be easily expanded to cover additional crops and pesticides.

## CONFLICT OF INTEREST

The authors declare no known competing financial interest.

## AUTHOR CONTRIBUTIONS

Nannan Pang & Xinze Liu: Conceptualization, Experiment, Writing. Peter Fantke: Conceptualization, Validation, Editing, Supervision. Qi Zhang, Liyuan Liu, Qiaozhen Chen & Qiyu Gong: Experiment and editing. Jiye Hu: Supervision, Editing.

## Supporting information


**Data S1.** Supporting Information.

## Data Availability

The data that supports the findings of this study are available in the supplementary material of this article.

## References

[1] Le Cor F , Slaby S , Dufour V , Iuretig A , Feidt C , Dauchy X *et al*., Occurrence of pesticides and their transformation products in headwater streams: contamination status and effect of ponds on contaminant concentrations. Sci Total Environ 788:147715 (2021). 10.1016/j.scitotenv.2021.147715. 34020090

[2] Steingrímsdóttir MM , Petersen A and Fantke P , A screening framework for pesticide substitution in agriculture. J Clean Prod 192:306–315 (2018). 10.1016/j.jclepro.2018.04.266.

[3] Ryberg MW , Rosenbaum RK , Mosqueron L and Fantke P , Addressing bystander exposure to agricultural pesticides in life cycle impact assessment. Chemosphere 197:541–549 (2018). 10.1016/j.chemosphere.2018.01.088. 29407816

[4] Kosnik MB , Hauschild MZ and Fantke P , Toward assessing absolute environmental sustainability of chemical pollution. Environ Sci Technol 56:4776–4787 (2022). 10.1021/acs.est.1c06098. 35349278 PMC9022439

[5] Kosnik MB , Antczak P and Fantke P , Data‐driven characterization of genetic variability in disease pathways and pesticide‐induced nervous system disease in the United States population. Environ Health Perspect 132:57003 (2024). 10.1289/EHP14108. 38752992 PMC11098008

[6] Gentil C , Fantke P , Mottes C and Basset‐Mens C , Challenges and ways forward in pesticide emission and toxicity characterization modeling for tropical conditions. Int J Life Cycle Assess 25:1290–1306 (2019). 10.1007/s11367-019-01685-9.

[7] Nemecek T , Antón A , Basset‐Mens C , Gentil‐Sergent C , Renaud‐Gentié C , Melero C *et al*., Operationalising emission and toxicity modelling of pesticides in LCA: the OLCA‐Pest project contribution. Int J Life Cycle Assess 27:527–542 (2022). 10.1007/s11367-022-02048-7.

[8] Gentil C , Basset‐Mens C , Manteaux S , Mottes C , Maillard E , Biard Y *et al*., Coupling pesticide emission and toxicity characterization models for LCA: application to open‐field tomato production in Martinique. J Clean Prod 277:124099 (2020). 10.1016/j.jclepro.2020.124099.

[9] Anastassiades M , Lehotay SJ , Štajnbaher D and Schenck FJ , Fast and easy multiresidue method employing acetonitrile extraction/partitioning and “dispersive solid‐phase extraction” for the determination of pesticide residues in produce. J AOAC Int 86:412–431 (2003). 10.1093/jaoac/86.2.412. 12723926

[10] Perestrelo R , Silva P , Porto‐Figueira P , Pereira JAM , Silva C , Medina S *et al*., QuEChERS‐fundamentals, relevant improvements, applications and future trends. Anal Chim Acta 1070:1–28 (2019). 10.1016/j.aca.2019.02.036. 31103162

[11] Pang NN , Cui Y and Hu JY , Weather dependent dynamics of the herbicides florasulam, carfentrazone‐ethyl, fluroxypyr‐meptyl and fluroxypyr in wheat fields through field studies and computational simulation. Chemosphere 165:320–328 (2016). 10.1016/j.chemosphere.2016.09.026. 27664521

[12] Hashimoto F , Takanashi H , Nakajima T , Ueda T , Kadokawa J , Ishikawa H *et al*., Occurrence of imidacloprid and its transformation product (imidacloprid‐nitroguanidine) in rivers during an irrigating and soil puddling duration. Microchem J 153:104496 (2020). 10.1016/j.microc.2019.104496.

[13] Nath R , Komala G , Fantke P and Mukherjee S , Dissipation kinetics, residue modeling and human intake of endosulfan applied to okra (*Abelmoschus esculentus*). Sci Total Environ 835:155591 (2022). 10.1016/j.scitotenv.2022.155591. 35490803

[14] Jacobsen RE , Fantke P and Trapp S , Analysing half‐lives for pesticide dissipation in plants. SAR QSAR Environ Res 26:325–342 (2015). 10.1080/1062936X.2015.1034772. 25948099

[15] Fantke P , Charles R , de Alencastro LF , Friedrich R and Jolliet O , Plant uptake of pesticides and human health: dynamic modelling of residues in wheat and ingestion intake. Chemosphere 85:1639–1647 (2011). 10.1016/j.chemosphere.2011.08.030. 21955352

[16] Fantke P , Juraske R , Antón A , Friedrich R and Jolliet O , Dynamic multicrop model to characterize impacts of pesticides in food. Environ Sci Technol 45:8842–8849 (2011). 10.1021/es201989d. 21905656

[17] Fantke P , Wieland P , Wannaz C , Friedrich R and Jolliet O , Dynamics of pesticide uptake into plants: from system functioning to parsimonious modelling. Environ Model Softw 40:316–324 (2013). 10.1016/j.envsoft.2012.09.016.

[18] Fantke P , Wieland P , Juraske R , Shaddick G , Sevigné‐Itoiz E , Friedrich R *et al*., Parameterization models for pesticide exposure via crop consumption. Environ Sci Technol 46:12864–12872 (2012). 10.1021/es301509u. 23136826

[19] Fantke P and Jolliet O , Life cycle human health impacts of 875 pesticides. Int J Life Cycle Assess 21:722–733 (2016). 10.1007/s11367-015-0910-y.

[20] Soheilifard F , Mark J , Zhang YY and Fantke P , Farm‐level environmental sustainability assessment of agricultural pest control strategies across Europe. Sustain Prod Consum 58:237–250 (2025). 10.1016/j.spc.2025.06.019.

[21] Mankong P , Fantke P , Phenrat T , Mungkalasiri J , Gheewala SH and Prapaspongsa T , Characterizing country‐specific human and ecosystem health impact and damage cost of agricultural pesticides: the case for Thailand. Int J Life Cycle Assess 27:1334–1351 (2022). 10.1007/s11367-022-02094-1.

[22] Mankong P , Fantke P , Ghose A , Soheilifard F , Oginah SA , Phenrat T *et al*., Assessing life cycle impacts from toxic substance emissions in major crop production systems in Thailand. Sustain Prod Consum 46:717–732 (2024). 10.1016/j.spc.2024.03.013.

[23] Pang NN , Fan XQ , Fantke P , Zhao SM and Hu JY , Dynamics and dietary risk assessment of thiamethoxam in wheat, lettuce and tomato using field experiments and computational simulation. Environ Pollut 256:113285 (2020). 10.1016/j.envpol.2019.113285. 31733956

[24] Feng XX , Wang K , PanL X , Xu TH , Zhang HY and Fantke P , Measured and modeled residue dynamics of famoxadone and oxathiapiprolin in tomato fields. J Agric Food Chem 66:8489–8495 (2018). 10.1021/acs.jafc.8b02056. 30028951

[25] Sevigné‐Itoiz E , Fantke P , Juraske R , Kounina A and Vallejo AA , Deposition and residues of azoxystrobin and imidacloprid on greenhouse lettuce with implications for human consumption. Chemosphere 89:1034–1041 (2012). 10.1016/j.chemosphere.2012.05.066. 22717159

[26] Juraske R , Fantke P , Ramírez ACR and González A , Pesticide residue dynamics in passion fruits: comparing field trial and modelling results. Chemosphere 89:850–855 (2012). 10.1016/j.chemosphere.2012.05.007. 22673401

[27] Feng XX , Pan LX , Xu TH , Jing J and Zhang HY , Dynamic modeling of famoxadone and oxathiapiprolin residue on cucumber and Chinese cabbage based on tomato and lettuce archetypes. J Hazard Mater 375:70–77 (2019). 10.1016/j.jhazmat.2019.04.075. 31048137

[28] Xiao SL , Li ZJ and Fantke P , Improved plant bioconcentration modeling of pesticides: the role of periderm dynamics. Pest Manag Sci 77:5096–5108 (2021). 10.1002/ps.6549. 34236751 PMC8518939

[29] Feng XX , Pan LX , Jing J , Zhang JC , Zhuang M , Zhang Y *et al*., Dynamics and risk assessment of pesticides in cucumber through field experiments and model simulation. Sci Total Environ 773:145615 (2021). 10.1016/j.scitotenv.2021.145615. 33582344

[30] Fantke P , Arnot JA and Doucette WJ , Improving plant bioaccumulation science through consistent reporting of experimental data. J Environ Manage 181:374–384 (2016). 10.1016/j.jenvman.2016.06.065. 27393944

[31] Fantke P and Juraske R , Variability of pesticide dissipation half‐lives in plants. Environ Sci Technol 47:3548–3562 (2013). 10.1021/es303525x. 23521068

[32] Zhang SY , Zhang Y , Ren SH , Lu HW , Li JM , Liang XY *et al*., Uptake, translocation and metabolism of acetamiprid and cyromazine by cowpea (*Vigna unguiculata* L.). Environ Pollut 331:121839 (2023). 10.1016/j.envpol.2023.121839. 37201568

[33] Liu QY , Liu YC , Dong FS , Sallach JB , Wu XH , Liu XG *et al*., Uptake kinetics and accumulation of pesticides in wheat (*Triticum aestivum* L.): impact of chemical and plant properties. Environ Pollut 275:116637 (2021). 10.1016/j.envpol.2021.116637. 33582637

[34] Wang WF , Wan Q , Li YX , Xu WJ and Yu XY , Uptake, translocation and subcellular distribution of pesticides in Chinese cabbage (*Brassica rapa var. chinensis*). Ecotoxicol Environ Saf 183:109488 (2019). 10.1016/j.ecoenv.2019.109488. 31376804

[35] Zhang JJ , Liu YC and Yang H , Chemical modification and degradation of atrazine in Medicago sativa through multiple pathways. J Agric Food Chem 62:9657–9668 (2014). 10.1021/jf503221c. 25226578

[36] Liu JN , Cheng JJ , Zhou CL , Ma LY , Chen XL , Li Y *et al*., Uptake kinetics and subcellular distribution of three classes of typical pesticides in rice plants. Sci Total Environ 858:159826 (2023). 10.1016/j.scitotenv.2022.159826. 36374729

[37] Felizeter S , McLachlan MS and Voogt PD , Uptake of perfluorinated alkyl acids by hydroponically grown lettuce (*Lactuca sativa*). Environ Sci Technol 46:11735–11743 (2012). 10.1021/es302398u. 23043263

[38] Li YB , Sallach JB , Zhang W , Boyd SA and Li H , Insight into the distribution of pharmaceuticals in soil‐water‐plant systems. Water Res 152:38–46 (2019). 10.1016/j.watres.2018.12.039. 30660096

