# Supplementary material for: Residue dynamics and dietary risks of diflufenican and flufenacet in wheat using field experiments and model simulations
Source: Pest Manag Sci. 2026 Mar 5;82(7):6207–17. doi: 10.1002/ps.70702 (PMC13240702; doi:10.1002/ps.70702)
Supplement: Supplementary file 1 — Data S1. Supporting Information. [file PS-82-6207-s001.docx]

**Supporting information**

Residue dynamics and dietary risks of diflufenican and flufenacet in wheat using field experiments and model simulations

Nannan Pang^a, #^, Xinze Liu^a, #^, Peter Fantke^b,c,d, *^, Qi Zhang^a^, Liyuan Liu^c^, Qiaozhen Chen^a^, Qiyu Gong^a^, Jiye Hu^a, *^

^a^ Lab of Pesticide Residues and Environmental Toxicology, School of Chemistry and Biological Engineering, University of Science and Technology Beijing, Beijing, 100083, PR China

^b^ substitute ApS, Graaspurvevej 55, 2400, Copenhagen, Denmark

^c^ Department for Evolutionary Ecology and Environmental Toxicology, Goethe University, 60438, Frankfurt am Main, Germany

^d^ Department of Environmental Sciences, College of Agriculture and Environmental Sciences, University of South Africa, Florida 1710, Roodepoort, South Africa

^e^ Guobiao (Beijing) Testing & Certification Co., Ltd, Beijing 101407, PR China

# The two authors contributed equally.

* Corresponding author (Peter Fantke [peter@substitute.dk](mailto:pefan@dtu.dk,), Jiye Hu jyhu@ustb.edu.cn)

**E-mail:** addresses: [sevensmiling@163.com (NN](mailto:sevensmiling@163.com%20(NN) Pang), [ustblxz@gmail.com (XZ](mailto:ustblxz@gmail.com%20(XZ) Liu), peter@substitute.dk (P Fantke), [zhangqi771105@163.com](mailto:zhangqi771105@163.com) (Q Zhang), [liuliyuan331@163.com (LY](mailto:liuliyuan331@163.com%20(LY), Liu), [42022076@xs.ustb.edu.cn](mailto:42022076@xs.ustb.edu.cn) (QZ Chen), [qyg18103549793@163.com](mailto:qyg18103549793@163.com) (QY Gong), [jyhu@ustb.edu.cn (J](mailto:jyhu@ustb.edu.cn%20(J)Y Hu).

There are 21 pages, 3 Figures and 8 Tables in all in this supporting material.

**S1 Properties and current studies of diflufenican and flufenacet**

Diflufenican (CAS NO. 83164-33-4) and flufenacet (CAS NO. 142459-58-3) were for the weed control (structures in Table S1). They belonged to phenyl ether and oxyacetamide herbicide. Diflufenican, N-(2,4-difluorophenyl)-2-[3-(trifluoromethyl)phenoxy]-3-pyridinecarboxamide is selectively and principally absorbed by shoots of germination seedlings. Diflufenican inhibits carotenoid biosynthesis and interferes indirectly with plant photosynthesis [1]. Flufenacet, N-(4-fluorophenyl)-N-(1-methylethyl)-2-[[5-(trifluoromethyl)-1,3,4-thiadiazol-2-yl]oxy]-acetamide has been found effectively against major annual grasses and certain small seeded dicotyledonous weeds [2, 3]. It is an oxyacetamide herbicide inhibiting very long chain fatty acid biosynthesis in plants [4]. In addition, it can be applied pre and early post emergence due to primary uptake via both roots and emerging shoots [5]. Due to their control capacity towards weed resistance, the combined composition has been welcomed by farmers. These pesticides have been used together for the weed control of cereals, corn, cotton, peanut and potato since the initial development in Bayer CropScience. Maximum residue limit (MRLs) of diflufenican and flufenacet are listed in Table S4 and in the range of 0.01-0.6 mg kg^-1^ in cereal. For crops such as wheat, rice and corn registered in China, the strictest MRL is issued by European Union (EU), namely 0.01 mg kg^-1^ in rice, among different legislation including China, Codex Alimentarius Commission (CAC), USA and EU. Up to now, although they are widely used, there is no MRL for diflufenican in USA and CAC and flufenacet in CAC yet. Literature have provided varied soil persistence, for example 60 d [6] and up to 90 d [7, 8]. Nowadays, the determination methodology for diflufenican and flufenacet has been updated from gas chromatography (GC)-electron capture detection [1, 6, 9] to QuEChERS with GC-MS [10-12] and LC-MS [13]. However, in those methods, diflufenican and flufenacet were just involved in large-scale screening studies without more detailed evaluation of these two specific pesticides, e.g. 171 pesticides [10], 167 pesticides [11], 120 pesticides [13] and 30 pesticides [12]. There is no report of their dissipation in crops, either. Their toxicities are also different according to the toxicity evaluation parameters such as acceptable daily intake (ADI) and acute reference dosage (ARfD) (Table S4). Their ADIs are 0.2 and 0.005 mg kg^-1^ bw, respectively. The ARfD of flufenacet is 0.017 mg kg^-1^ bw. As a result, flufenacet is more toxic. Overall, despite of their wide usage, the information about their determination, dissipation, residue and dietary risk assessment has been insufficient. Considering co-occurrence, persistence, and potential environmental and health hazard, it is necessary to compare their dissipation, residue distribution and risk evaluation of these two pesticides.

**S2 The calculation of dietary risk assessments for pesticides**

For pesticides, there are both long term and acute dietary risk assessment to obtain the exposure risk of humans. For the long-term evaluation, the average national individual daily intake (NEDI) estimates the long-term average intake of the specific pesticide per person. It is the sum of the appropriate residue in all intaking food, following Equation S1 as follow.

$NEDI =\sum_{i}(\mathrm{STMR}\times F )/ body weight$ (S1)

Where STMR_i_ (mg kg^-1^) is the standard median residue representing the appropriate residue value; i represents different crops; F_i_ is the consumption amount of a given food by a person of the specific human group; body weight (kg) is the average value of a person in the human group. If taking China as an example, the average body weight is 63 kg for a Chinese adult; the food intake (Fi) is 138.5 g. Usually, STMR_i_ in the crop is obtained from field trials. In some safe cases, the corresponding MRL can be used for the calculation to maximize the risk, because STMR_i_ is at least not larger than MRL.

Long-term evaluation pays attention to the chronic risk quotient, namely, the ratio between NEDL and ADI, following Equation S2.

RQ_C_ = NEDI / ADI × 100% (S2)

Where RQ_C_ represents chronic or long-term risk quotient. If RQ_C_ <100, the long-term risk of the pesticide is acceptable for human.

Similarly, for the acute evaluation, the average national individual short-term intake (NESTI) estimates the acute maximum intake of the specific pesticide. Different foods have their unit weight (Ue) (if existed), the variability factor for intake correction (with large Ue) and the large portion (LP) (97.5% of the daily consumption) of food intake in one meal.

$NESTI = \left\{ \begin{aligned} \frac{HR\times\mathrm{LP}}{body weight} if no Ue \\ \frac{\mathrm{Ue}\times HR\times\upsilon+(LP-Ue)P\times HR}{body weight} if Ue<LP \\ \frac{HR\times\mathrm{LP}\times\upsilon}{body weight} if Ue>LP \end{aligned} \right.$ (S3)

Where HR is the highest residue in field trials. Some food such as apples etc. have Ue. Some food such as watermelon with large Ue also consider their variability factors for intake correction. Most food such as wheat in this work only need to consider the large portion in one meal for human. For example, the wheat LP is 732.96 g /person for Chinese general population (https://www.who.int/teams/nutrition-and-food-safety/databases/global-environment-monitoring-system-food-contamination). In acute dietary assessment, body weights include two types, namely 16.1 kg for children (1-6 years old) and 63 kg for the general population (> 1 years). The acute risk quotient for short term intake can be calculated by Equation S4.

RQa = NESTI / ARfD × 100% (S4)

Where RQ_a_ represents acute risk quotient. If RQ_a_ < 100, the short-term risk of the pesticide is acceptable for human.

**S3 The established QuEChERS LC-MS/MS method**

QuEChERS involves extraction as well as cleanup by dispersive solid phase extraction. As mentioned in previous application [14], QuEChERS modified with acid buffers can enhance recoveries of pH-sensitive pesticides. In this study, based on experience and optimization, acid buffer of 2% acetic acid in acetonitrile and subsequent 4 g MgSO_4_ and 1 g NaCl provided satisfactory salting out extraction efficiency. In addition, simple and common sorbents of 50 mg PSA and 100 mg MgSO_4_ provided acceptable cleanup effect. A typical chromatogram was shown in Figure S1.

Recovery is one of the most important parameters in pesticides residue analysis. In present study, different fortified levels of 0.01, 0.1 and 10 mg kg^-1^ were chosen for soil, straw, and grain. Figure S2 showed the recovery data for diflufenican and flufenacet in three matrices. The repeatability and trueness of the method were studied by carrying out five consecutive extractions of spiked matrices. The recoveries in all test levels (n=5) were in the range of 85 to 103% (91-101% for soil, 88-102% for wheat grain, and 85-103% for straw) (Figure S2). *RSDs* were below 10% for all cases (1.6-6.9% for soil, 1.9-9.9% for wheat grain, and 1.2-8.7% for straw).

Although it sometimes improves the signal of targets, matrix effects in LC-MS mostly and greatly deteriorate the LC-MS method’s sensitivity. It is closely related to the specific target as well as the type of matrices. Such is an anxiety that either isotopically labeled standards or matrix matched calibration can be used to circumvent and compensate the situation [15]. In this work, the latter was much cheaper and easier and employed to illustrate the change of ionization efficiency in the presence of other compounds. The matrix effect is expressed as the ratio of the slope of standards in matrix and the slope in solvent. And the range of 80-120% is acceptable. As shown in Table S3, suppression of analyte response varied considerably from matrix to matrix and differed substantially with matrix effects of 72.7% to 109%. It further illustrated the present QuEChERS could provide satisfactory cleanup effects.

As shown in Table S3, quantitation including the linear regression equations relating the concentration to area using the established QuEChERS LC-MS and the LOQ of each analyte were acceptable, together with correlation coefficients (*R*^2^) higher than 0.99. The method repeatability was satisfactory due to *RSDs* lower than 2.9% in different matrices including soil, wheat grain, and straw. Besides, the LOQ of each analyte was 0.01 mg kg^-1^ in different matrices.

Altogether, a versatile QuEChERS LC-MS method with simple and common sorbents has been established for diflufenican and flufenacet in three matrices, with acceptable quantitation, satisfactory recoveries and LOQs.

**References**

[1] E Wennberg, L Torstensson. Gas-chromatographic methods for determination of diflufenican in soil. International Journal of Environmental Analytical Chemistry, 1997, 67(1-4), 73-79. https://doi.org/10.1080/03067319708031395

[2] R Deege, H Foerster, RR Schmidt, W Thielert, MA Tice, GJ Aadesen, HJ Santel. BAY FOE 5043: a new low rate herbicide for pre-emergence grass control in corn, cereals, soyabeans and other selected crops. Proc Brighton Crop Protection Conference Weeds, 1995, 1, 43-48.

[3] H Förster, RR Schmidt, HJ Sante, R Andree. FOE 5043-a new selective herbicide from the oxyacetamide group. Pflanzenschutz-Nachrichten Bayer English Edition, 1997, 50, 105-116.

[4] SG Kleemann, P Boutsalis, GS Gill, C Preston. Applications of pre-emergent pyroxasulfone, flufenacet and their mixtures with triallate for the control of Bromus diandrus (ripgut brome) in no-till wheat (*Triticum aestivum*) crops of southern Australia. Crop Protection, 2016, 80, 144-148. https://doi.org/10.1016/j.cropro.2015.11.010

[5] WJ Grichar, BA Besler, KD Brewer, DT Palrang. Flufenacet and metribuzin combinations for weed control and corn (*Zea mays*) tolerance. Weed technology, 2003, 17(2), 346-351. [https://doi.org/10.1614/0890-037X(2003)017[0346:FAMCFW]2.0.CO;2](https://doi.org/10.1614/0890-037X(2003)017%5b0346:FAMCFW%5d2.0.CO;2)

[6] J Rouchaud, O Neus, R Bulcke, K Cools, H Eelen, T Dekkers. Soil dissipation of diuron, chlorotoluron, simazine, propyzamide, and diflufenican herbicides after repeated applications in fruit tree orchards. Archives of Environmental Contamination and Toxicology, 2000, 39(1), 60-65. <https://doi.org/10.1007/s002440010080>

[7] S Gupta, VT Gajbhiye. Effect of concentration, moisture and soil type on the dissipation of flufenacet from soil. Chemosphere, 2002, 47(9), 901-906. <https://doi.org/10.1016/S0045-6535(02)00017-6>

[8] J Rouchaud, O Neus, K Cools, R Bulcke. Flufenacet soil persistence and mobility in corn and wheat crops. Bulletin of Environmental Contamination and Toxicology, 1999, 63(4), 460-466. [https://doi.org/10.1007/s001289901002](https://doi.org/10.1080/02772248.2016.1196209)

[9] L Patty, C Guyot. Analytical methods for the determination of isoproturon and diflufenican residues in runoff and soil. Bulletin of Environmental Contamination and Toxicology, 1995, 55(6), 802-809.

[10] YT Han, L Song, N Zou, R Chen, YH Qin, CP Pan. Multi-residue determination of 171 pesticides in cowpea using modified QuEChERS method with multi-walled carbon nanotubes as reversed-dispersive solid-phase extraction materials. Journal of Chromatography B-Analytical Technologies in the Biomedical and Life Sciences, 2016, 1031, 99-108. <https://doi.org/10.1016/j.jchromb.2016.07.043>

[11] S Walorczyk, D Drożdżyński. Improvement and extension to new analytes of a multi-residue method for the determination of pesticides in cereals and dry animal feed using gas chromatography-tandem quadrupole mass spectrometry revisited. Journal of Chromatography A, 2012, 1251, 219-231. <http://doi.org/10.1016/j.chroma.2012.06.055>

[12] PY Zhao, L Wang, L Zhou, FZ Zhang, S Kang, CP Pan. Multi-walled carbon nanotubes as alternative reversed-dispersive solid phase extraction materials in pesticide multi-residue analysis with QuEChERS method. Journal of Chromatography A, 2012, 1225, 17-25. <https://doi.org/10.1016/j.chroma.2011.12.070>

[13] P Kaczyński. Large scale multi-class herbicides analysis in oilseeds by rapid one-step QuEChERS-based extraction and cleanup method using liquid chromatography-tandem mass spectrometry. Food Chemistry, 2017, 230, 411-422. <http://doi.org/10.1016/j.foodchem.2017.03.076>

[14] NN Pang, TL Wang, JY Hu, BZ Dong. Field evaluation and determination of four herbicides in a wheat ecosystem by a simple and versatile QuEChERS method with liquid chromatography-tandem mass spectrometry. Toxicological & Environmental Chemistry, 2017, 99(3), 376-389. https://doi.org/10.1080/02772248.2016.1196209

[15] NN Pang, TL Wang, JY Hu. Method validation and dissipation kinetics of four herbicides in maize and soil using QuEChERS sample preparation and liquid chromatography tandem mass spectrometry. Food Chemistry, 2016, 190, 793-800. https://doi.org/10.1016/j.foodchem.2015.05.081

**Table S1** Physicochemical properties, degradation half-lives and applied amount in the model and experiment for diflufenican and flufenacet.

| Compound | diflufenican | flufenacet |
| --- | --- | --- |
| CAS NO. | 83164-33-4 | 142459-58-3 |
| structure |  |  |
| molecular weight  (g mol^-1^) | 394.29 | 363.33 |
| octanol-water partition coefficient  (L kg^-1^) | 3.2×10^3^ | 2.0×10^2^ |
| air-water partition coefficient | 9.9×10^-5^ | 2.4×10^-6^ |
| t_1/2_ ^a^ soil (d) in model | 140 ^b^ | 32 ^c^ |
| Mapplied in model (kg m^-2^) | 1 kg m^-2^ | |
| Mapplied in experiment  (mg m^-2^) | 0.2 | 0.4 |
| MRL^d^ (mg kg^-1^) in the model | 0.05 (rice) ^e^ | 0.5 (rice) ^f^ |

^a^ Half-lives

^b^ J Rouchaud, O Neus, R Bulcke, K Cools, H Eelen, T Dekkers. Soil dissipation of diuron, chlorotoluron, simazine, propyzamide, and diflufenican herbicides after repeated applications in fruit tree orchards. Archives of Environmental Contamination and Toxicology, 2000, 39(1), 60-65. <https://doi.org/10.1007/s002440010080>

^c^ J Rouchaud, O Neus, K Cools, R Bulcke. Flufenacet soil persistence and mobility in corn and wheat crops. Bulletin of Environmental Contamination and Toxicology, 1999, 63(4), 460-466. https://doi.org/10.1007/s001289901002

^d^ MRL= maximum residue limits

^e^ MRL in EU

^f^ MRL in China

**Table S2** Multi-reaction monitoring condition of LC-MS/MS for diflufenican and flufenacet

| Analytes | structure | retention time (min) | Transition  (*m/z*) | quant ^a^  qual ^b^ | collision energy  (V) | fragmentor (V) | mode |
| --- | --- | --- | --- | --- | --- | --- | --- |
| diflufenican |  | 1.5 | 395-266  395-246 | 266  246 | 24  26 | 120 | positive |
| flufenacet |  | 1.0 | 364-152  364-194 | 152  194 | 15  4 | 105 | positive |

^a^ quantitative ion

^b^ qualitative ion

**Table S3** LOQs, linearity and repeatability (n=6) for determination of diflufenican and flufenacet in different matrices

| Pesticides | sample | regression equation ^a^ | linear range  (μg mL^-1^) | correlation coefficient (*R*^2^) | *RSD*% ^b^  (n=6) | LOQ ^c^  (mg kg^-1^) | matrix effect (%) |
| --- | --- | --- | --- | --- | --- | --- | --- |
| diflufenican | acetonitrile | y = 5.4×10^4^x + 1.6×10^4^ | 0.005~5 | 0.9981 | 0.41 |  |  |
|  | soil | y = 5.0×10^4^x + 1.6×10^4^ | 0.005~5 | 0.9975 | 0.40 | 0.01 | 93.1 |
|  | wheat grain | y = 3.9×10^4^x + 9.8×10^3^ | 0.005~5 | 0.9983 | 2.9 | 0.01 | 72.7 |
|  | straw | y = 4.6×10^4^x + 1.1×10^4^ | 0.002~5 | 0.9984 | 1.4 | 0.01 | 84.0 |
| flufenacet | acetonitrile | y = 5.8×10^4^x + 6.2×10^3^ | 0.005~5 | 0.9998 | 1.1 |  |  |
|  | soil | y = 6.4×10^4^x + 2.0×10^4^ | 0.005~5 | 0.9978 | 0.48 | 0.01 | 109 |
|  | wheat grain | y = 5.7×10^4^x + 1.6×10^4^ | 0.005~5 | 0.9983 | 0.82 | 0.01 | 97.3 |
|  | straw | y = 4.7×10^4^x + 6.8×10^3^ | 0.002~5 | 0.9995 | 0.22 | 0.01 | 80.5 |

^a^ y = area; x = concentration (μg mL^-1^)

^b^ *RSD* = relative standard deviation

^c^ LOQ = limits of quantification

**Table S4** Maximum residue levels (MRLs), acceptable daily intake (ADIs) and acute reference dosage (ARfD) of diflufenican and flufenacet

| Compounds | registered crops | food classification | MRLs | | | | ADI  mg kg bw^-1^ | ARfD  mg kg bw^-1^ |
| --- | --- | --- | --- | --- | --- | --- | --- | --- |
|  |  |  | China | CAC^a^ | USA | European Union |  |  |
| diflufenican | rice | cereal |  |  |  | 0.01 ^b^ | 0.2 | - ^c^ |
|  | wheat |  | 0.05 |  |  | 0.02 |  |  |
| flufenacet | wheat | cereal | 0.5 |  | 0.60 | 0.1 | 0.005 | 0.017 |
|  | corn |  | 0.05 |  | 0.05 | 0.05 |  |  |

^a^ CAC= Codex Alimentarius Commission

^b^ Temporary MRL

^c^ Toxicity is low

**Table S5** Dissipate kinetics and half-lives of diflufenican and flufenacet in wheat ecosystem under field conditions

| Compounds | matrix | site ^a^ | regression equation ^b^ | half-life  (d) | initial deposit  (mg kg^-1^) | correlation coefficient  (*R*^2^) |
| --- | --- | --- | --- | --- | --- | --- |
| diflufenican | straw | A | Ct= 3.3exp(-0.094t) | 7.4 | 4.2 | 0.8773 |
|  |  | B | Ct= 5.6exp(-0.43t) | 16 | 7.1 | 0.8943 |
|  |  | C | Ct= 10exp(-0.084t) | 8.3 | 10 | 0.9819 |
|  | soil | A | Ct= 1.2exp(-0.051t) | 14 | 1.1 | 0.9734 |
|  |  | B | Ct= 2.6exp(-0.064t) | 11 | 0.48 | 0.9166 |
|  |  | C | Ct= 0.44exp(-0.016t) | 43 | 2.3 | 0.8367 |
| flufenacet | straw | A | Ct= 6.0exp(-0.21t) | 3.3 | 12 | 0.9430 |
|  |  | B | Ct= 7.5exp(-0.40t) | 1.7 | 6.4 | 0.9445 |
|  |  | C | Ct= 16exp(-0.27t) | 2.6 | 17 | 0.9673 |
|  | soil | A | Ct= 2.2exp(-0.071t) | 9.8 | 2.2 | 0.9932 |
|  |  | B | Ct= 5.5exp(-0.068t) | 10 | 0.74 | 0.8966 |
|  |  | C | Ct= 0.69exp(-0.018t) | 38 | 4.4 | 0.9250 |

^a^ Site A = Beijing of China (116.46°E, 39.92°N); Site B = Shandong Province of China (120.42°E, 36.52°N); Site C = Anhui of China (116.93°E, 34.19°N)

^b^ *C_t_* = residue level (mg kg^-1^); *t* = time (day)

**Table S6** Wheat straw residues of diflufenican and flufenacet in field trials

| Compound | time interval | wheat straw residue levels (n=3)(dissipation %^a^) | | |
| --- | --- | --- | --- | --- |
|  |  | site A^b^ | site B^c^ | site C^d^ |
| diflufenican | 0（2h） |  |  |  |
|  | 1 d | 1.2±6.3 | 35.6±1.3 | 20.3±2.8 |
|  | 3 d | 29.9±5.1 | -27.3±4.3 | 12.2±2.8 |
|  | 5 d | 37.1±7.7 | 0.0±10.6 | 10.4±3.8 |
|  | 7 d | 64.0±1.4 | 40.3±3.8 | -0.4±2.6 |
|  | 14 d | 56.6±4.2 | 60.9±1.5 | 49.2±1.9 |
|  | 21 d | 47.4±1.9 | -148±41.8 | 59.0±1.4 |
|  | 30 d | -17.8±13.8 | 49.4±7.7 | 36.6±11.1 |
| flufenacet | 0（2h） |  |  |  |
|  | 1 d | 14.7±0.7 | 46.2±0.5 | 28.8±0.3 |
|  | 3 d | 37.2±0.6 | 64.5±0.1 | 62.8±0.4 |
|  | 5 d | 55.2±0.2 | 6.5±0.5 | 20.9±0.5 |
|  | 7 d | 87.5±0.1 | 48.0±0.3 | -2.7±0.3 |
|  | 14 d | 80.8±0.2 | 89.6±0.1 | 82.6±0.0 |
|  | 21 d | 72.6±0.5 | 41.4±0.5 | 94.0±0.2 |
|  | 30 d | 0.0±0.0 | 70.0±1.4 | 74.2±1.0 |

^a^ dissipation%=（1-residue at time t/residue at 0 d）

^b^ site A= Beijing (116.46°E, 39.92°N)

^c^ site B= Shandong (120.42°E, 36.52°N)

^d^ site C= Anhui (116.93°E, 34.19°N)

**Table S7** Modelled parameters in spatial and temporal distribution of diflufenican and flufenacet in wheat ecosystem

| Parameters |  | diflufenican | flufenacet |
| --- | --- | --- | --- |
| t_m, max_^a^/d | leaf | 0.1 | 0.25 |
|  | grain | 20 | 13 |
|  | stem | 0 | 5 |
|  | root | 17 | 1 |
| residence time  t^b^/d | air | 2.16 | 0.48 |
|  | soil | 19.08 | 6.16 |
|  | leaf surface | 1.72 | 3.34 |
|  | grain surface | 11.90 | 12.07 |
|  | leaf | 5.66 | 9.22 |
|  | grain | 16.27 | 15.66 |
|  | stem | 11.39 | 2.08 |
|  | root | 4.47 | 0.87 |
| hF_max_^c^  /kg _harvest_ kg _applied_^-1^ | leaf | 0.56 | 0.56 |
|  | grain | 5.7E-2 | 5.4E-2 |
|  | stem | 0.29 | 2.1E-2 |
|  | root | 1.7E-2 | 6.6E-3 |
| iF_max_^d^  /kg _intake_ kg _applied_^-1^ | grain | 1.9E-2 | 1.8E-2 |

^a^ t_m, max_: maximal mass of pesticides occurring time in plants (d)

^b^ t: residence time of pesticides in plants (d)

^c^ hF_max_: maximal mass of pesticides in plants (kg _harvest_ kg _applied_^-1^)

^d^ iF_max_: maximal mass in humans *via* ingestion of harvest (kg _intake_ kg _applied_^-1^)

**Table S8** Chronic dietary risk assessment of diflufenican and flufenacet for different human groups

| Pesticide | registered  crop classification | ADI  mg kg bw^-1^ | age | sex | cereal intake  g | body  weight  kg | modelled | | | field | | |
| --- | --- | --- | --- | --- | --- | --- | --- | --- | --- | --- | --- | --- |
|  |  |  |  |  |  |  | STMR^b^  mg kg^-1^ | NEDI^c^  mg kg bw^-1^ | RQc^d^  % | STMR^b^  mg kg^-1^ | NEDI^c^  mg kg bw^-1^ | RQc^d^  % |
| diflufenican | cereal  (wheat, rice) | 0.2 | 2-7 | children | 218.3 | 17.9 | 0.011 | 0.0024 | 0.067 | 0.01 | 0.0022 | 0.061 |
|  |  |  | 8-12 | children | 336 | 33.1 |  | 0.0037 | 0.056 |  | 0.0034 | 0.051 |
|  |  |  | 13-19 | male | 461.8 | 56.4 |  | 0.0051 | 0.045 |  | 0.0046 | 0.041 |
|  |  |  | 13-19 | female | 368.8 | 50 |  | 0.0041 | 0.041 |  | 0.0037 | 0.037 |
|  |  |  | 20-50 | male | 475.7 | 63 |  | 0.0052 | 0.042 |  | 0.0048 | 0.038 |
|  |  |  | 20-50 | female | 386.9 | 56 |  | 0.0043 | 0.038 |  | 0.0039 | 0.035 |
|  |  |  | 51-65 | male | 461 | 65 |  | 0.0051 | 0.039 |  | 0.0046 | 0.035 |
|  |  |  | 51-65 | female | 391.8 | 58 |  | 0.0043 | 0.037 |  | 0.0039 | 0.034 |
|  |  |  | ≥65 | male | 398.8 | 59.5 |  | 0.0044 | 0.037 |  | 0.0040 | 0.034 |
|  |  |  | ≥65 | female | 326.3 | 52 |  | 0.0036 | 0.035 |  | 0.0033 | 0.031 |
| flufenacet | cereal  (wheat, corn) | 0.005 | 2-7 | children | 218.3 | 17.9 | 0.022 | 0.0048 | 5.37 | 0.01 | 0.0022 | 2.44 |
|  |  |  | 8-12 | children | 336 | 33.1 |  | 0.0074 | 4.47 |  | 0.0034 | 2.03 |
|  |  |  | 13-19 | male | 461.8 | 56.4 |  | 0.0102 | 3.60 |  | 0.0046 | 1.64 |
|  |  |  | 13-19 | female | 368.8 | 50 |  | 0.0081 | 3.25 |  | 0.0037 | 1.48 |
|  |  |  | 20-50 | male | 475.7 | 63 |  | 0.0105 | 3.32 |  | 0.0048 | 1.51 |
|  |  |  | 20-50 | female | 386.9 | 56 |  | 0.0085 | 3.04 |  | 0.0039 | 1.38 |
|  |  |  | 51-65 | male | 461 | 65 |  | 0.0101 | 3.12 |  | 0.0046 | 1.42 |
|  |  |  | 51-65 | female | 391.8 | 58 |  | 0.0086 | 2.97 |  | 0.0039 | 1.35 |
|  |  |  | ≥65 | male | 398.8 | 59.5 |  | 0.0088 | 2.95 |  | 0.0040 | 1.34 |
|  |  |  | ≥65 | female | 326.3 | 52 |  | 0.0072 | 2.76 |  | 0.0033 | 1.26 |

^a^ ADI = acceptable daily intake (mg kg^-1^)

^b^ STMR = supervised trials median residue levels

^c^ NEDI = average national estimated individual daily intake (NEDI)

^d^ RQc = chronic risk quotient

**Figure S1** Typical chromatogram of diflufenican and flufenacet by QuEChERS LC-MS

**Figure S2** Recoveries of diflufenican and flufenacet in different matrices including soil, wheat grain, and straw with different fortified levels

**
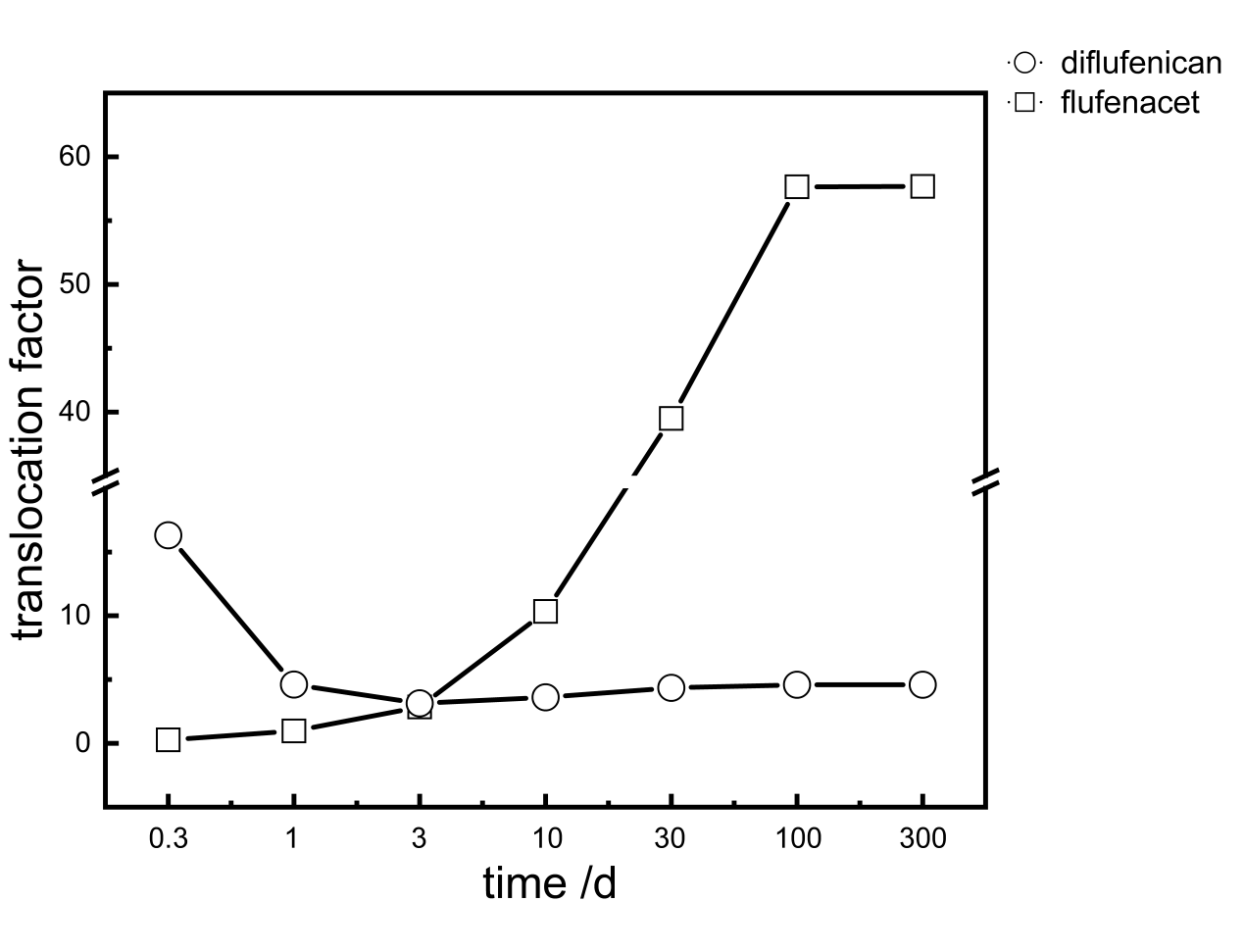
**

**Figure S3** Translocation factors of diflufenican and flufenacet in wheat
